# Supplementary material for: Photosensitizer-specific bacterial stress responses in Escherichia coli reveal distinct targets in photoinduced inactivation
Source: Commun Biol. 2025 Oct 1;8:1413. doi: 10.1038/s42003-025-08881-4 (PMC12488859; doi:10.1038/s42003-025-08881-4)
Supplement: Supplementary file 1 — Supplementary Information [file 42003_2025_8881_MOESM1_ESM.docx]

**Supporting information**

**Photosensitizer-Specific Bacterial Stress Responses in *Escherichia coli* Reveal Distinct Targets in Photoinduced Inactivation**

**Marco Chittò^1,2^, David Tutschner^1^, Ulrich Dobrindt^1,2^, Anzhela Galstyan^3*^, Michael Berger^1*^**

^1^Institute of Hygiene, University of Münster, Münster, Germany

^2^AO Research Institute Davos, Davos, 7270, Switzerland

^3^ University of Duisburg-Essen, Faculty of Chemistry, Center for Nanointegration Duisburg Essen (CENIDE), Center for Water and Environmental Research (ZWU) and Center for Molecular Biotechnology (ZMB), Universitätsstrasse 5, 45141 Essen, Germany

*For correspondence

**Supplementary material and methods**

*General information*

The bacterial strains used in this study are listed in Table S 1. All the plasmids used in this study are listed in Table S 2. All the primers used in this study were purchased from Sigma-Aldrich^®^ (Taufkirchen, Germany) and are listed in Table S 3. All the restriction enzymes and the T4 DNA ligase were purchased from New England Biolabs (Frankfurt, Germany). The relevant parts of all constructs (chromosome-reporter module junctions as well as promoter-reporter gene junctions) were Sanger sequenced after analytical PCR for the chromosomal constructs and analytical restriction digestion for the plasmid constructs.

**Table S 1**. List of bacterial strains used in this study

| ***E. coli* strains** | **Reference** | |
| --- | --- | --- |
| *E. coli* K-12 DH5α | | Taylor *et al*., 1993 |
| *E. coli* K-12 MG1655 | | Blattner *et al*., 1996 |
| *E. coli* K-12 MG1655 pPS2 | | Schiller *et al*., 2021 |
| *E. coli* K-12 MG1655 Δ*rpoS* pPS2 | | Schiller *et al*., 2021 |
| *E. coli* K-12 MG1655 pWKS30 | | This study |
| *E. coli* K-12 MG1655 *att::*P*frr*-*cfp-aph(3’)‐Ia* | | Lang et al 2022 |
| *E. coli* K-12 MG1655 P*gadA*-*yfp-cat* | | This study |
| *E. coli* K-12 MG1655 P*spy*-*yfp-cat* | | This study |
| *E. coli* K-12 MG1655 P*pspA*-*yfp-cat* | | This study |
| *E. coli* K-12 MG1655 P*otsA*-*yfp-cat* | | This study |
| *E. coli* K-12 MG1655 *att::*P*frr*-*cfp-cat-yfp-*P*gadA* | | This study |
| *E. coli* K-12 MG1655 *att::*P*frr*-*cfp-cat-yfp-*P*spy* | | This study |
| *E. coli* K-12 MG1655 *att::*P*frr*-*cfp-cat-yfp-*P*otsA* | | This study |
| *E. coli* K-12 MG1655 *att::*P*frr*-*cfp-cat-yfp-*P*recA* | | Lang *et al.*, 2022 |
| *E. coli* K-12 MG1655 *att::*P*frr*-*cfp-cat-yfp-*P*dps* | | This study |
| *E. coli* K-12 MG1655 *att::*P*frr*-*cfp-cat-yfp-*P*dps oxyR::aph* | | This study |
| *E. coli* K-12 MG1655 *att::*P*frr*-*cfp-cat-yfp-*P*dps mntR::aph* | | This study |
| *E. coli* K-12 MG1655 *att::*P*frr*-*cfp-cat-yfp-*P*spy baeR::aph* | | This study |
| *E. coli* K-12 MG1655 *att::*P*frr*-*cfp-cat-yfp-*P*spy cpxR::aph* | | This study |

**Table S 2**. Oligonucleotides used in this study

| **Primer** | **Sequence (5’→3’)** | |
| --- | --- | --- |
| MBP5 | | GGATGAATGGCAGAAATTCG |
| MBP206 | | GCTGAACTTGTGGCCGTTTA |
| MBP279 | | ggcgcaatgccatctggtat |
| MBP280 | | gacgggaaactgaaaatgtg |
| MBPD155 | | CTCTCGACCGCTCTGCCTT |
| MC162 | | CCAGCTGTTTTTTTAAAGGCTGGGCATTCGGTTTTTACAACGTTATGTTAAGGAAACAGCTATGACCATG |
| MC163 | | GGATAAATCCTACTTTTTTATTGCCTTCAAATAAATTTAAGGAGTTCGAAATGGTGTCTATCACTAAAGA |
| MC164 | | TGTGTGATGTTCTACGGGCA |
| MC166 | | CTGCAAGGTAGTGGACAAGACCGGCGGTCTTAAGTTTTTTGGCTGAAAGAAGGAAACAGCTATGACCATG |
| MC167 | | ACGCTACTTAAGAAAGCCGTAATAAATAACTGAAAGGAAGGATATAGAATATGGTGTCTATCACTAAAGA |
| MC168 | | CGGCCCGTTCTTTTCATTGT |
| MC178 | | CAAGTGAGGTCGATGTGCTGTTAGTTCCACTTACGGGAGATTAACCGCTCAGGAAACAGCTATGACCATG |
| MC179 | | TAAAGCGCGTTCTGCGCAACACAATAAGAAAAGAGAAGGAGGAGAACCGGATGGTGTCTATCACTAAAGA |
| MC180 | | ATCAATTAGCTGCGCCGATG |
| MC183 | | AACTTTTTGTCTTTTTACCTTCCCGTTTCGCTCAAGTTAGTATAAAAAAGATTTACATTTCTGCATGGTT |
| MC185 | | AGGAAACAGCTATGACCATGATTAC |
| MC187 | | TAAATGCTTCAATAATATTGAAAAAGGAAGAGTATGAGTATTCAACATGCAGGAAACAGCTATGACCATG |
| MC191 | | CAACTTTTTGTCTTTTTACCTTCCCGTTTCGCTCAAGTTAGTATAAAAAATTAATTTGATCGCCCGAACA |
| MC194 | | CAACTTTTTGTCTTTTTACCTTCCCGTTTCGCTCAAGTTAGTATAAAAAACCCGCAGAAATCATTCTGCG |
| MC197 | | CAACTTTTTGTCTTTTTACCTTCCCGTTTCGCTCAAGTTAGTATAAAAAATGCTTTTTTCTGCAACAATT |
| MC206 | | GTTTGGGCGATTTTTATTACG |
| MC207 | | CCAGTCATCCGGTATAGTTC |
| MC209 | | CGGTTGGCTGTTCTTCGTTG |
|  | |  |
|  | |  |
|  |  | |
|  |  | |

**Table S 3**. List of plasmids used in this study

| **Plasmid** | **Reference** |
| --- | --- |
| pKD46 | Datsenko and Wanner., 2000 |
| pKD4 | Datsenko and Wanner., 2000 |
| pPS2 | Schiller *et al*., 2021 |
| pMB54 | Berger *et al*., 2016 |

**Figure S 1**. Viability of *E.coli* K-12 MG1655 under the same conditions used in this study (15 min irradiation, 660 nm ± 26 nm, 10 mW/cm2, 9 J/cm2).


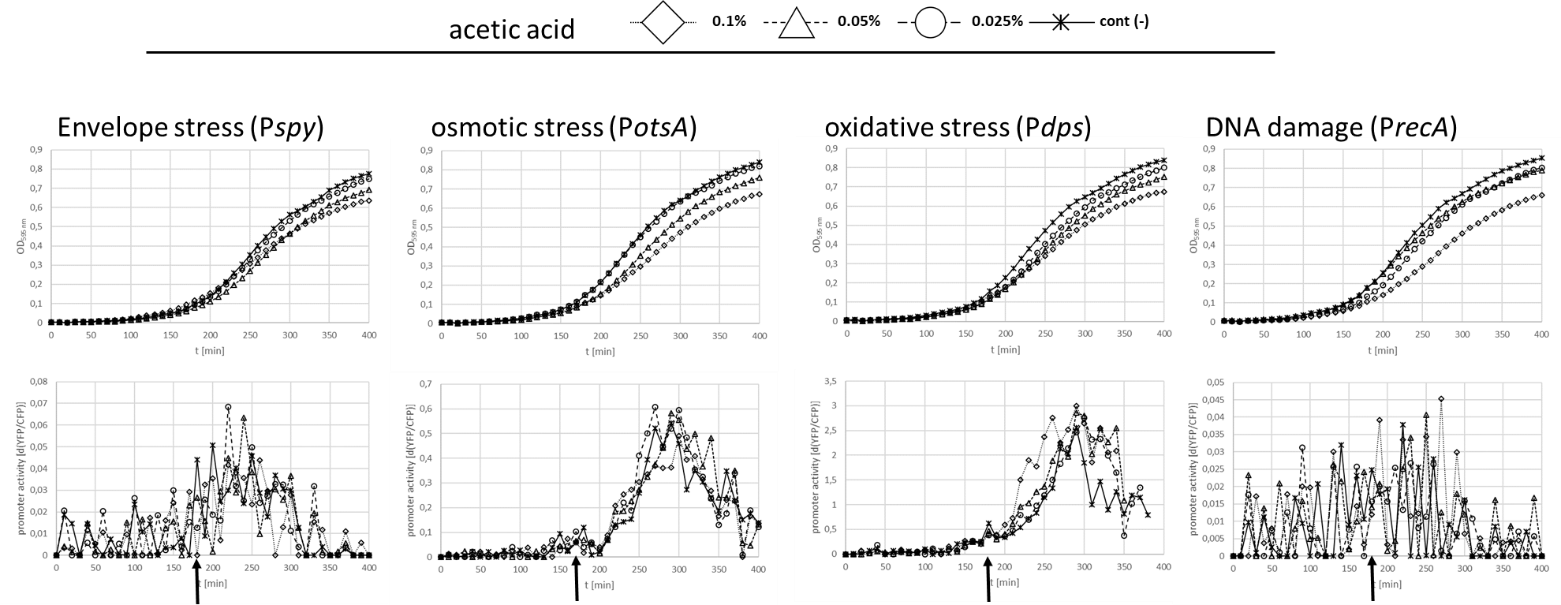


**Figure S 2. Response of the stress reporter modules to different concentrations of acetic acid.** Shown are growth curves of the reporter strain (top) and the response of the indicated reporter module response over time (bottom). A black arrow indicates the time point of acetic acid addition. For the details, see the text.

**
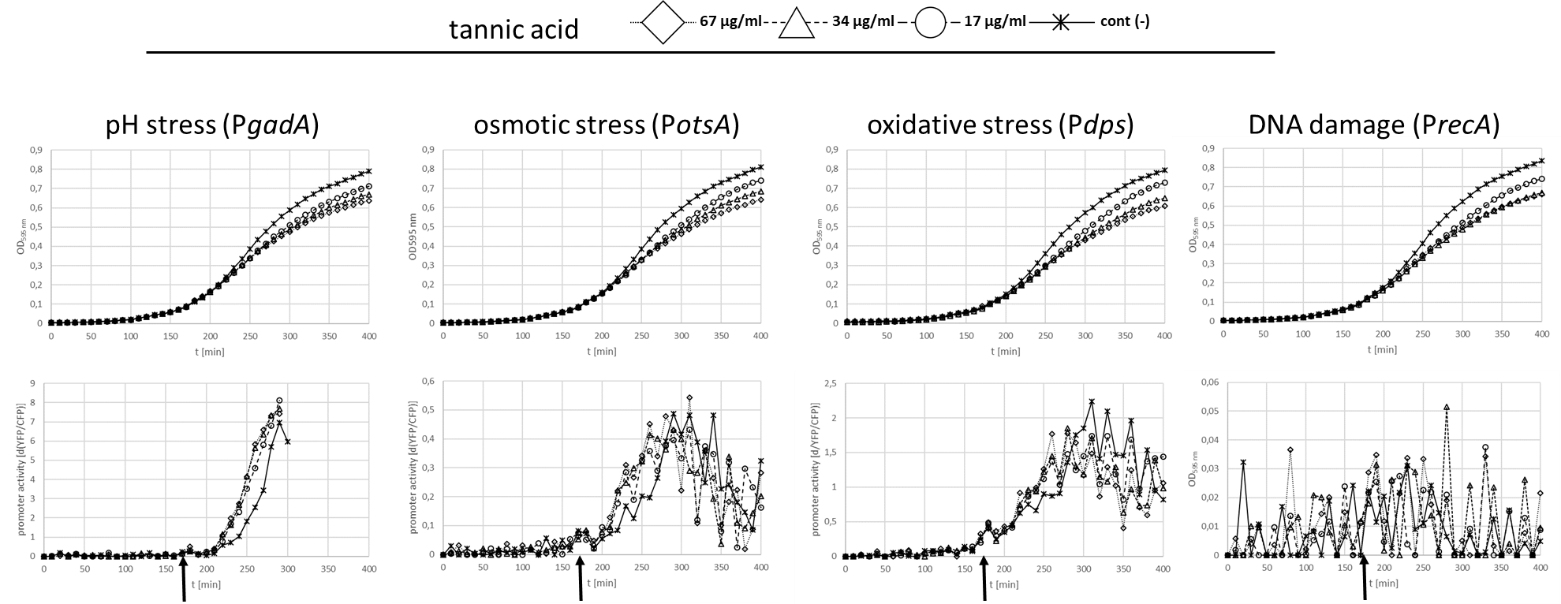
**

**Figure S 3. Response of the stress reporter modules to different concentrations of tannic acid.** Shown are growth curves of the reporter strain (top) and the response of the indicated reporter module response over time (bottom). A black arrow indicates the time point of tannic acid addition. For the details, see the text.

**
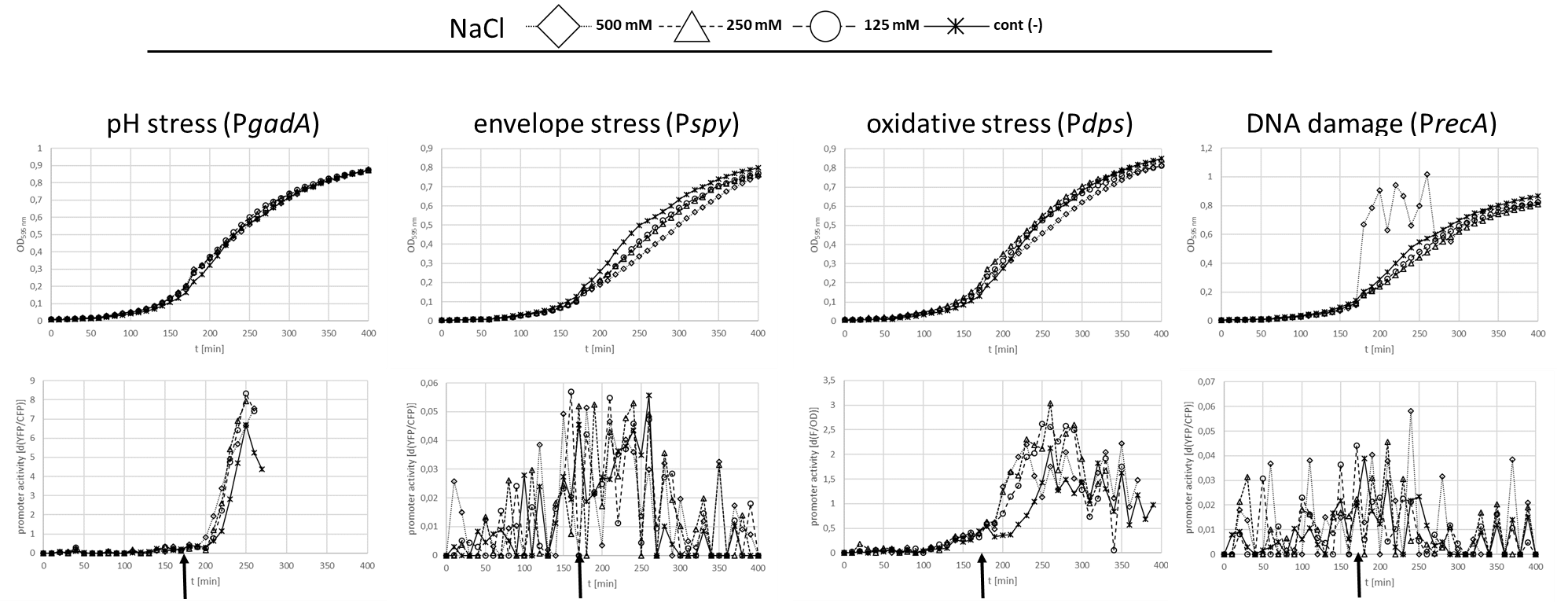
**

**Figure S 4. Response of the stress reporter modules to different concentrations of sodium chloride (NaCl).** Shown are growth curves of the reporter strain (top) and the response of the indicated reporter module response over time (bottom). A black arrow indicates the time point of NaCl. For the details, see the text.

**
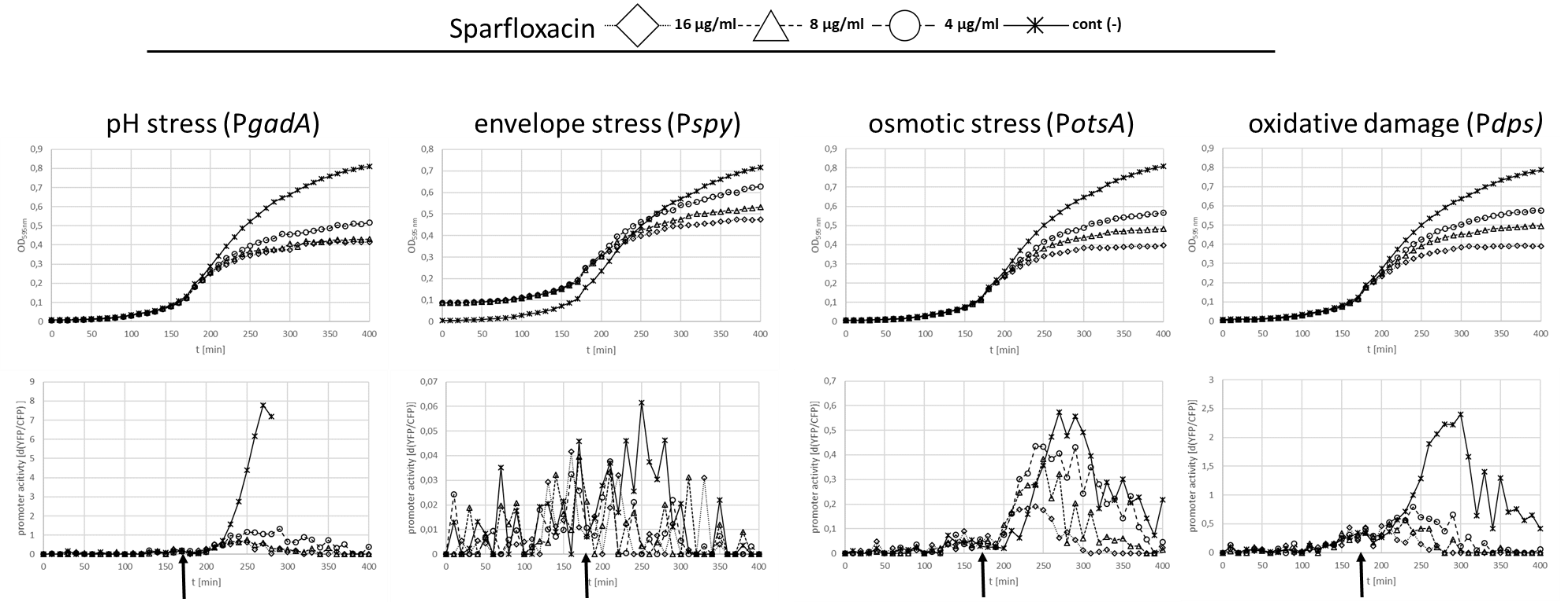
**

**Figure S 5. Response of the stress reporter modules to different concentrations of sparfloxacin.** Shown are growth curves of the reporter strain (top) and the response of the indicated reporter module response over time (bottom). A black arrow indicates the time point of sparfloxacin. For the details, see the text.

**
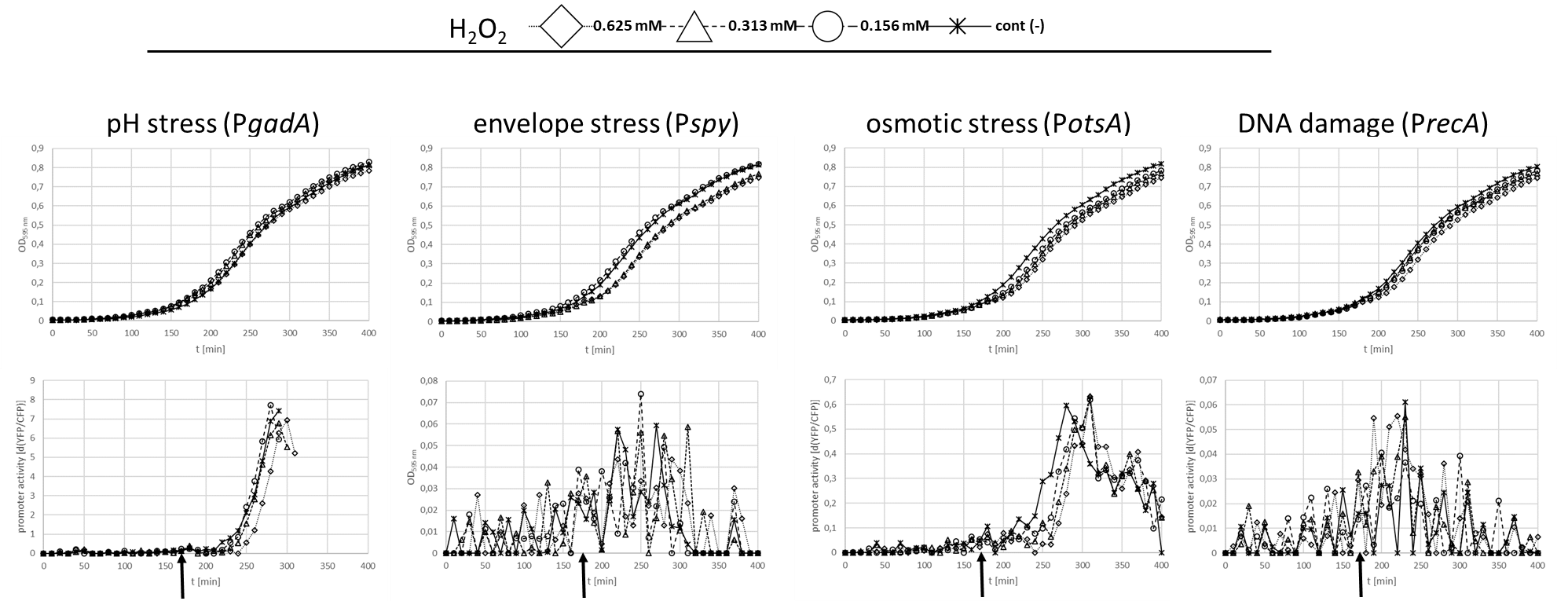
**

**Figure S 6. Response of the stress reporter modules to different concentrations of H_2_O_2_.** Shown are growth curves of the reporter strain (top) and the response of the indicated reporter module response over time (bottom). A black arrow indicates the time point of H_2_O_2_. For the details, see the text.

**
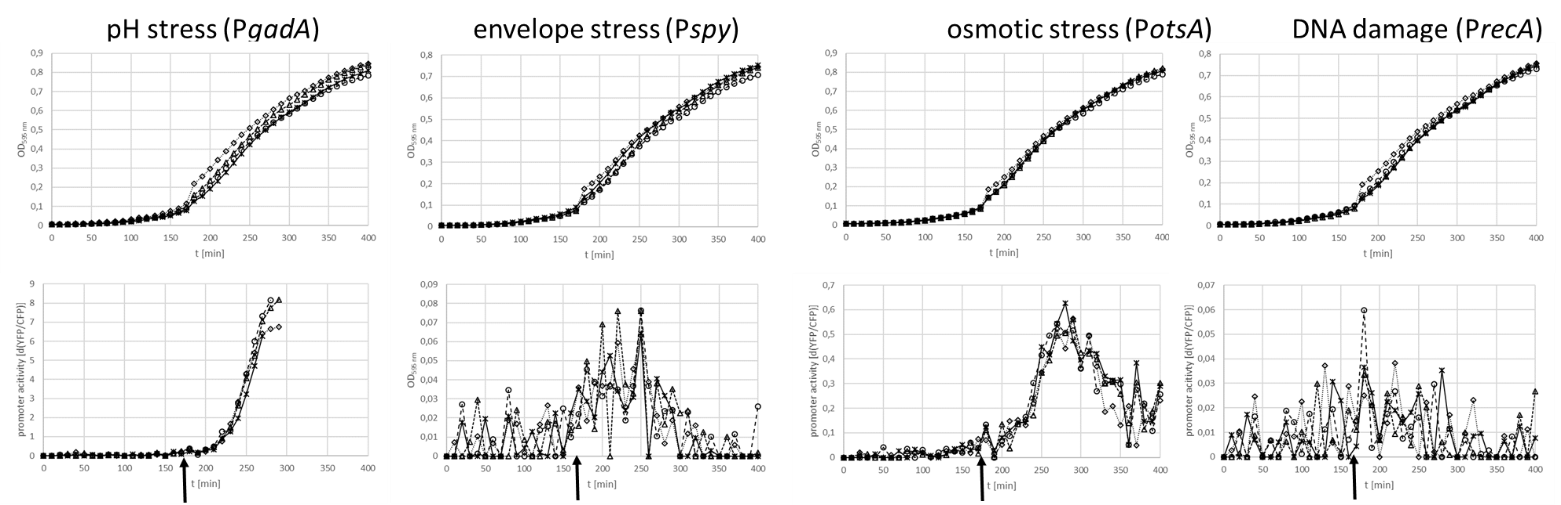
**

**Figure S 7. Response of the stress reporter modules to different concentrations to MB (dark).** Shown are growth curves of the reporter strain (top) and the response of the indicated reporter module response over time (bottom). A black arrow indicates the time point of MB. For the details, see the text.

**
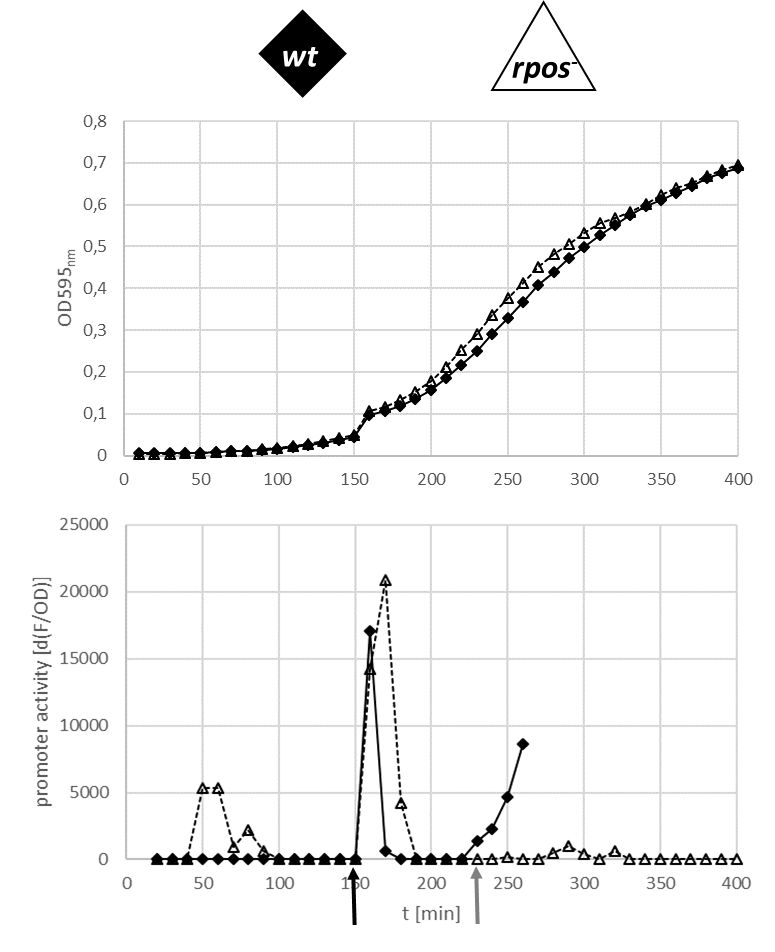
**

**Figure S 8. The use of *rpoS* mutants facilitates the detection MB dependent response of the *dps* promoter.** Shown are the growth curves (top) and the response of the *dps* promoter (bottom) in *E. coli* K-12 MG1655 pPS2 (black diamonds) and *E. coli* K-12 MG1655 *rpoS^-^* pPS2 (empty triangles) to 5 µM MB after light activation (black arrow). The grey arrow indicates the RpoS dependent growth phase activation of the *dps* promoter that is absent in the *rpoS* mutant.

**
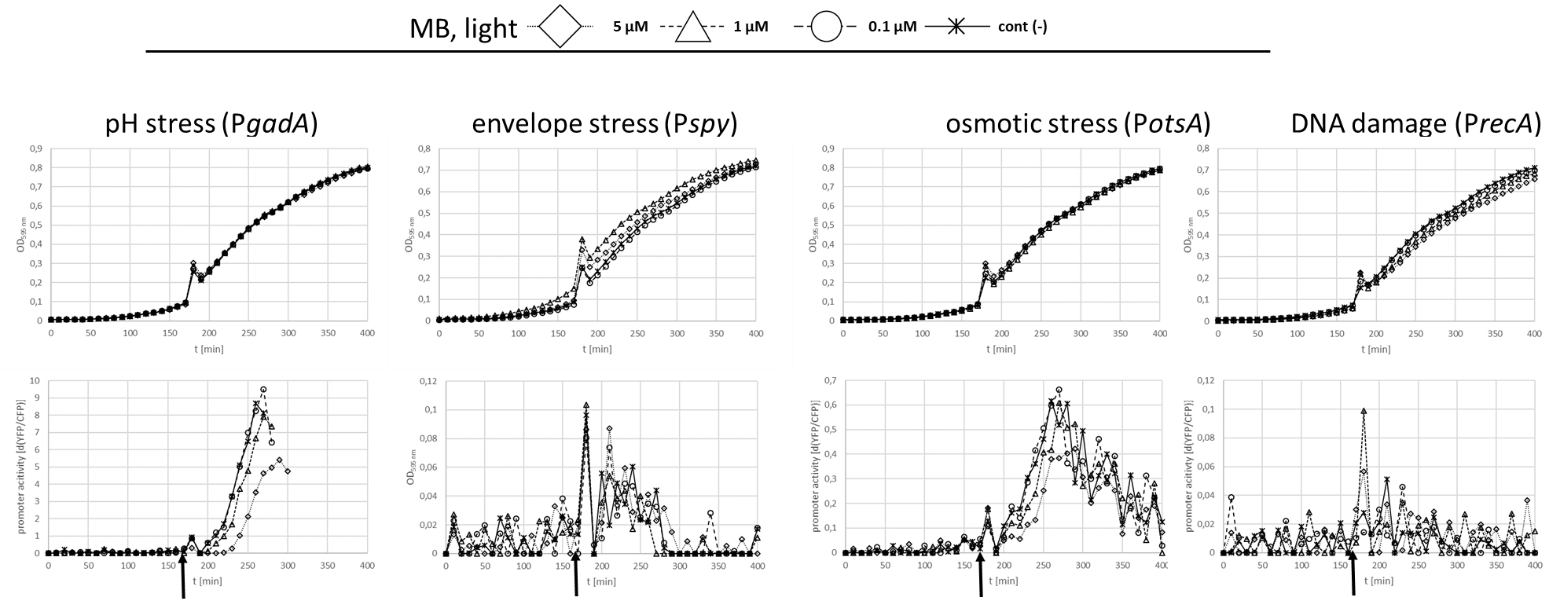
**

**Figure S 9. Response of the stress reporter modules to different concentrations to MB (light).** Shown are growth curves of the reporter strain (top) and the response of the indicated reporter module response over time (bottom). A black arrow indicates the time point of MB that was followed by 15 min exposure to light (660 nm). The control condition labeled “cont (–)” represents cells treated with water and exposed to light, serving as the light-only control. For further experimental details, please refer to the main text.

**
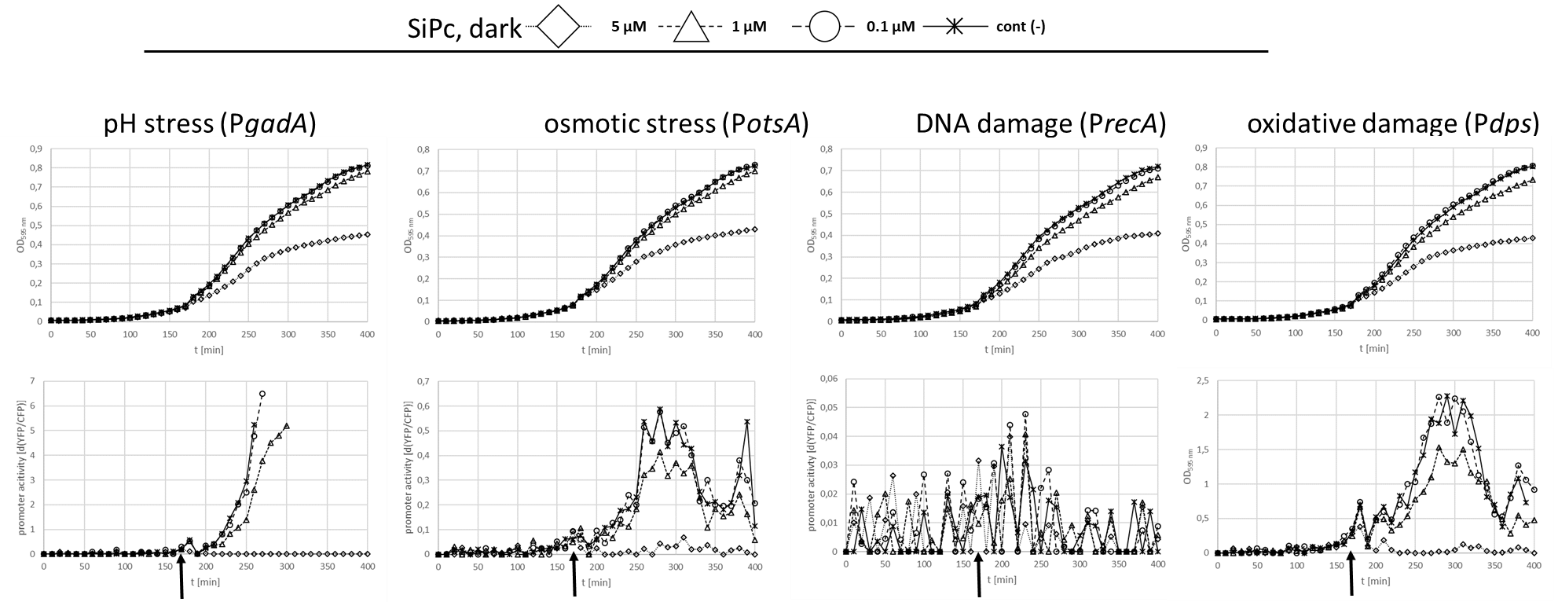
**

**Figure S 10. Response of the stress reporter modules to different concentrations to SiPc(dark).** Shown are growth curves of the reporter strain (top) and the response of the indicated reporter module response over time (bottom). A black arrow indicates the time point of SiPc. The control condition labeled “cont (–)” represents cells treated with water and exposed to light, serving as the light-only control. For further experimental details, please refer to the main text.

**
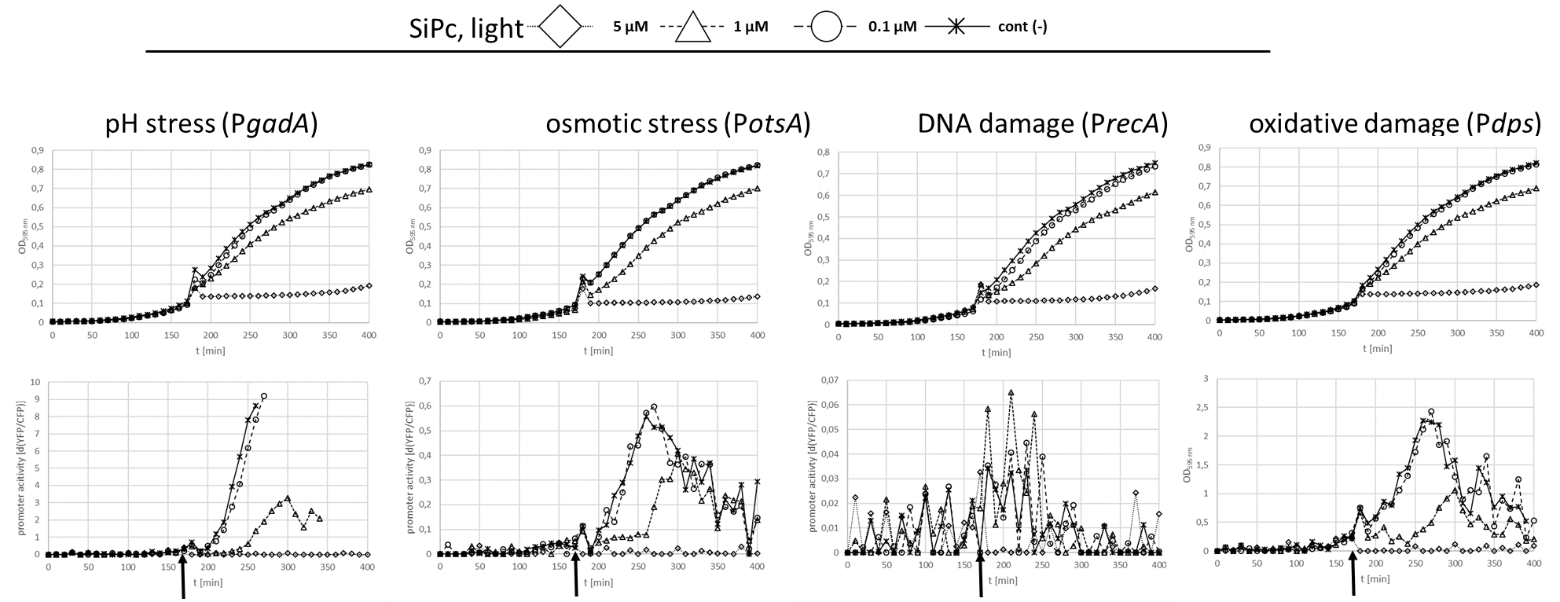
**

**Figure S 11. Response of the stress reporter modules to different concentrations to SiPc (light).** Shown are growth curves of the reporter strain (top) and the response of the indicated reporter module response over time (bottom). A black arrow indicates the time point of SiPc that was followed by 15 min exposure to light (660 nm). For the details, see the text.
