# Supplementary material for: Photosensitizer-specific bacterial stress responses in Escherichia coli reveal distinct targets in photoinduced inactivation
Source: Commun Biol. 2025 Oct 1;8:1413. doi: 10.1038/s42003-025-08881-4 (PMC12488859; doi:10.1038/s42003-025-08881-4)
Supplement: Supplementary file 2 — Description of Additional Supplementary Materials [file 42003_2025_8881_MOESM2_ESM.pdf]

Description of Additional Supplementary Files

File name: Supplementary Data 1

Description: The source data for figures 3-7
